# Open carry

**DOI:** 10.1080/19336934.2025.2503624

**Published:** 2025-05-21

**Authors:** Howy Jacobs

**Affiliations:** Faculty of Medicine and Health Technology, Tampere University, Finland

The recent case of a Harvard scientist being detained at the US border and ending up incarcerated in a facility for asylum seekers should be extremely alarming to all of us in the research community. For details of the affair, as known at the time of writing, please refer to the news item published in *The Guardian* [1]. Dr Kseniia Petrova, author of an important recent work in vertebrate developmental genetics [2], was initially refused entry at Boston airport due to a technicality regarding biological samples she was carrying. It seems that her visa was then revoked and she was transferred to the custody of the US Immigration and Customs Enforcement agency (ICE), after requesting not to be returned to her native country, Russia, on the grounds that she believed that she faced political persecution there.

Without getting into the legal niceties or the underlying political issues, which are themselves very serious, there is a clear lesson for all of us in the life sciences community, when travelling across international borders for academic or scientific purposes: make sure you have every possible document validating your identity and the purpose of your visit, and that you have fulfilled all legal requirements for the import or export of anything you are carrying, however inane, intrusive or nonsensical they may seem.

In the *Drosophila* world this applies particularly to the transport of living flies. Apart from the issues connected with animal welfare, to which *Drosophila* research is immune in most countries, more workaday matters need to be considered. For instance, we all have our in-house recipe for fly food. Depending on what’s in yours, the contents of a food vial may look suspiciously like a block of cannabis resin to a customs officer. In the past I usually just armed students or collaborators with a letter on headed notepaper, stating that they were carrying non-hazardous material for biological research. Sometimes I added the rider that these were fruit-flies of the species *Drosophila melanogaster*, which were completely harmless. But, over time, the word ‘fruit-fly’ came to be regarded by some officials as synonymous with ‘thermonuclear device’, and I eventually dropped it completely; no doubt leaving too much to chance. Burying something deep in your dirty underwear might work in some cases, but if discovered it is a *prima facie* admission of guilt.

Long ago, well before the days of tariffs, trade barriers and CT scanners, I fell victim to such a snare when travelling between Nice and Genoa to catch a cheap flight back to Glasgow. This was also long before the Schengen agreement. The Italian border agent refused me entry on the grounds that I wasn’t carrying a permit issued by an approved veterinary authority, for the biological samples that I was proposing to bring into the country, albeit for only an hour or two. I was obliged to catch the next train back and travel home from Nice airport instead, at an exorbitant last-minute fare.

Another obvious problem is the use of dry ice for carrying frozen materials, which is strictly forbidden in aircraft cabins – one of those prohibited items covered by the box you have to tick when checking in, listed on that page which nobody ever reads. My advice would be never to carry any scientific materials, perhaps even including data, across international frontiers. Though the only alternative is typically to use courier services at iniquitously high prices. To send flies I used to employ just the old fashioned post office, but even this is insecure and probably illegal in most countries. Nowadays you need to dig out the rules, read them thoroughly and in fine detail, find a courier able to deliver, make an honest and complete declaration and pay up. It’s far better than risking being sent to a detention centre for an unlimited time, housed in a dormitory with 80 other unlucky souls, and from which there may be no appeal, escape or redress.

Just being open about what you are carrying may not help. On one occasion when travelling between country A and country B I was proudly wearing my studded punk neckband. Yet, a customs officer of country B confiscated it on the grounds that it was ‘an undeclared weapon’. This despite the fact that nearly identical items were freely on sale nationwide in boutiques and record shops.

A year later, the science and innovation attaché at the local embassy of country B wrote to me at my university address to invite my participation in an international grants scheme for scientific collaboration with country C, where I was then located. I wrote back that I would be very happy to apply for such funding, but only if the authorities in country B were able to locate and return my seized property (worth maybe about 2 €). Amazingly, the item, which presumably had been carefully stored in some airport customs warehouse for 12 months – perhaps as some kind of trophy – was duly returned to me by overnight courier. But had it been flies, even the hardiest strain isolated and lovingly cultured by the world’s most prolific ageing researchers would have already succumbed long before [3].

The documents you carry may themselves arouse suspicion. For many years my only university ID was just a blank white plastic card with an embedded chip readable only by the device on my lab building’s entry doors: not very convincing when presented to an immigration officer. On one occasion, when entering country A to meet a collaborator and attend an international symposium that we had jointly organized, I had taken the precaution of having my host write a formal letter of invitation in hard copy. But when I presented it for scrutiny, the entry official at the secondary inspection to which I was directed was very sceptical, asking me rather unapprovingly if my relationship ‘with this Professor XXXXX’ was of a purely professional nature.

The global climate for science and scholarship is unfortunately darkening. Countries that were once joined by ties of kinship and shared values seem to have drifted into mutual suspicion or even open hostility. Scientists seem to be among the first casualties. Bureaucratic regulations that were once buried in a drawer are increasingly used as a tool for harassing innocent scholars, even if they achieve little more than keeping those who enforce them in employment. For now, our only option seems to be buckle down and make sure that we abide scrupulously by those regulations, however inane they may seem, so as to avoid getting caught up in an Orwellian nightmare. In truth, we have very little leverage other than the support of public opinion, which is itself increasingly dominated by unsympathetic voices. We can but hope for more enlightened times.
